# Letter from Elgin

**Published:** 1883-06

**Authors:** H. Rosencrans

**Affiliations:** Elgin


### Domestic Correspondence.

ARTICLE VIII.

ELGIN, MAY 3, 1883.

EDITORS MEDICAL JOURNAL AND EXAMINER:

Mrs. M., æt. thirty-five years; a widow for several years; never had any children. This lady several months ago applied to me for treatment for a uterine difficulty. Her history was that for nine years she had been undergoing medical treatment from several different physicians of the place, continually growing worse, and at last pronounced incurable. I examined the womb with the speculum, and found the organ considerably enlarged and retroverted, the fundus lying in Douglas' pouch. The mouth of the uterus was as nearly closed as possible. It was with difficulty that I could introduce the smallest-sized probe. She complained of a great deal of pain in the lower part of her back. Her catamenial flow was on her a good portion of the time, as often as every two weeks, and between these spells she would have a show of sanious discharge. My treatment of the case was to dilate the mouth of the uterus and restore the organ to its normal position. This I succeeded in doing with Ellinger's dilater and a uterine sound. I had to use the dilater several times, at intervals of two weeks, to permanently open the os uteri. This constituted the principal local treatment. The constitutional treatment was nutritious food, rest, ergot and iron, with occasional doses of potassic bromide. Cured.

H. ROSENCRANS, M.D.

